# Linking Neurobehavioral Symptoms to Productive Activities in Post-9/11 Veterans: A Correlational Analysis Using TVMI Data

**DOI:** 10.1093/milmed/usaf462

**Published:** 2025-10-09

**Authors:** Kelsee M Stromberg, Erin D Bouldin, Dawne Vogt, Shannon R Miles, Megan E Vanneman, Thomas N Maloney, Jacob Kean, Angela P Presson, Mary Jo Pugh

**Affiliations:** Department of Internal Medicine, Division of Epidemiology, Spencer Fox Eccles School of Medicine, University of Utah, 295 Chipeta Way, Salt Lake City, UT 84108, United States; Veterans Affairs Informatics, Decision-Enhancement, and Analytic Sciences (IDEAS) Center, VA Salt Lake City Health Care System, 30 N 1900 E, Salt Lake City, UT 84132, United States; Department of Internal Medicine, Division of Epidemiology, Spencer Fox Eccles School of Medicine, University of Utah, 295 Chipeta Way, Salt Lake City, UT 84108, United States; Veterans Affairs Informatics, Decision-Enhancement, and Analytic Sciences (IDEAS) Center, VA Salt Lake City Health Care System, 30 N 1900 E, Salt Lake City, UT 84132, United States; VA Boston Health Care System, 150 South Huntington Avenue, Boston, MA 02130, United States; Division of Psychiatry, Boston University Chobanian & Avedisian School Medicine, 72 East Concord Street, Boston, MA 02118, United States; Mental Health and Behavioral Sciences Services, James A. Haley Veterans’ Hospital, 13000 Bruce B. Downs Boulevard Tampa, FL 33612-4745, United States; Department of Neurosurgery, Brain and Spine, Morsani College of Medicine, University of South Florida, Tampa, Florida, 2 Tampa General Circle, STC 7th Floor, Tampa, FL 33606, United States; Department of Internal Medicine, Division of Epidemiology, Spencer Fox Eccles School of Medicine, University of Utah, 295 Chipeta Way, Salt Lake City, UT 84108, United States; Veterans Affairs Informatics, Decision-Enhancement, and Analytic Sciences (IDEAS) Center, VA Salt Lake City Health Care System, 30 N 1900 E, Salt Lake City, UT 84132, United States; Department of Population Health Sciences, University of Utah Spencer Fox Eccles School of Medicine, Salt Lake City, 295 Chipeta Way, Salt Lake City, Utah 84018, United States; Health System Innovation & Research (HSIR) Division, University of Utah, 295 Chipeta Way, Salt Lake City, Utah 84018, United States; Department of Internal Medicine, Division of Epidemiology, Spencer Fox Eccles School of Medicine, University of Utah, 295 Chipeta Way, Salt Lake City, UT 84108, United States; Department of Internal Medicine, Division of Epidemiology, Spencer Fox Eccles School of Medicine, University of Utah, 295 Chipeta Way, Salt Lake City, UT 84108, United States; Health System Innovation & Research (HSIR) Division, University of Utah, 295 Chipeta Way, Salt Lake City, Utah 84018, United States; Department of Internal Medicine, Division of Epidemiology, Spencer Fox Eccles School of Medicine, University of Utah, 295 Chipeta Way, Salt Lake City, UT 84108, United States; Department of Internal Medicine, Division of Epidemiology, Spencer Fox Eccles School of Medicine, University of Utah, 295 Chipeta Way, Salt Lake City, UT 84108, United States; Veterans Affairs Informatics, Decision-Enhancement, and Analytic Sciences (IDEAS) Center, VA Salt Lake City Health Care System, 30 N 1900 E, Salt Lake City, UT 84132, United States

## Abstract

**Introduction:**

Military veterans are at risk for long-term neurobehavioral symptoms who may hinder participation in productive activities following their transition out of military service. This study examined the association between injury-related neurobehavioral symptoms and engagement in paid and unpaid productive activities among post-9/11 veterans.

**Materials and Methods:**

This secondary cross-sectional analysis utilized data from the Veterans Metrics Initiative (TVMI) Study. Veterans who served in the U.S. military after September 11, 2001, and were within 90 days of separation from active duty—or from activated status in the National Guard or Reserve—were identified in Fall 2016 through records from the Department of Veterans Affairs/Department of Defense Identity Repository. Independent variables included self-reported vestibular, somatosensory, cognitive, and affective symptoms from the Defense and Veterans Brain Injury Center (DVBIC) survey. The dependent variable was engagement in productive activity, classified as: neither paid nor unpaid labor (reference), paid labor only, paid and unpaid labor, and unpaid labor only.

The relationship between neurobehavioral symptoms and productive activity status was assessed using multinomial logistic regression. In **Model 1,** we adjusted for pre-military traumatic brain injury (TBI) history, probable deployment TBI status, and demographic characteristics. **Model 2** added a positive screen for possible post-traumatic stress disorder (PTSD). Results are presented as relative risk ratios (RR), which represent the ratio of the probability of an outcome occurring in an exposed group to the probability of it occurring in a reference group.

**Results:**

Among 8,945 veterans (mean age = 35.7 years), 37.0% engaged in paid labor only, 21.4% in both paid and unpaid labor, 27.0% in unpaid labor only, and 14.6% in neither. Symptom prevalence was somatosensory (18.0%), affective (16.9%), cognitive (11.5%), and vestibular (7.2%). In Model 1, vestibular symptoms were linked to lower likelihood of engaging in paid labor only (RR = 0.54, 95% CI [0.40-0.74], *P* < .001) and both paid and unpaid labor (RR = 0.69, 95% CI [0.49-0.96], *P* = .027). Cognitive symptoms were also associated with a lower likelihood of paid labor only (RR = 0.67, 95% CI [0.49-0.91], *P* = .011). In Model 2 (adjusting for demographics and probable PTSD), vestibular symptoms remained significant (RR = 0.59, 95% CI [0.43-0.81], *P* = .001), although cognitive symptoms were no longer associated. Post-traumatic stress disorder emerged as a strong predictor with veterans screening positive being 53% less likely to engage in paid labor only (RR = 0.47, 95% CI [0.39-0.56], *P* < .001) and 37% less likely to engage in both paid and unpaid labor (RR = 0.63, 95% CI [0.52-0.76], *P* < .001).

**Conclusions:**

Vestibular and cognitive symptoms were related to less engagement in productive activities post-service for Veterans, with an emphasis on activities that included paid employment. Participation may improve with the treatment of neurobehavioral symptoms.

After leaving the military, veterans often face the task of reintegrating into civilian society by finding work, enrolling in school, or engaging in other productive roles. Although employment is an important component of reintegration, many veterans may choose to participate in unpaid activities such as furthering their education, engaging in volunteer work, or assuming caregiving responsibilities for family members, which we define as productive activity in alignment with the Mayo-Portland Adaptability Inventory (MPAI).^1^ The MPAI recognizes both paid employment and unpaid structured roles—including education, volunteering, caregiving, and homemaking—as valid forms of participation.^1^ It also emphasizes that the value and time commitment associated with a role, rather than financial compensation, determine its classification as productive.^1^

These roles beyond employment have been linked to improved quality of life and well-being in veteran populations.^2^^,^^3^ Pursuing higher education funded by the GI Bill allows veterans to gain new skills that may improve long-term employment prospects,^2^ although volunteer work enables veterans to contribute to their communities, socialize, and apply skills gained during military service to civilian environments.^3^ A study by Matthieu et al. 2017, found that veterans participating in a national civic service initiative experienced significant improvement in mental health and social outcomes, including reduced post-traumatic stress disorder (PTSD) symptoms, decreased isolation, enhanced self-efficacy, and greater perceived social support.^3^ Similarly, findings from the National Health and Resilience in Veterans Study suggest that veterans often view caregiving as a “rewarding experience”.^4^

Although neurobehavioral symptoms are frequently associated with traumatic brain injury (TBI), especially among post-9/11 veterans, they can also result from a variety of other medical, psychological, and environmental factors. Neurobehavioral symptoms—such as dizziness, memory issues, impulsivity, and fatigue—are relatively common among post-9/11 veterans, defined as individuals who served in the U.S. active military, naval, air, or space service after September 11, 2001, and who were discharged or released therefrom under conditions other than dishonorable.^5^

Traumatic brain injury which has been defined as the signature injury of the recent wars,^6–8^ is a well-documented contributor to neurobehavioral sequelae in this population. Although most individuals with mild TBI recover within 3 months,^9^ some continue to have chronic neurobehavioral symptoms, which may interfere with reintegration into civilian society.^9^^,^^10^ However, neurobehavioral symptoms can also arise from other conditions.^9–11^ Chronic pain, mental health disorders (e.g., PTSD, depression, and anxiety),^12–15^ neurological conditions, general aging-related changes, and prolonged stress exposure^11–13^^,^^16^ can all contribute to similar symptoms. For instance, vestibular dysfunction may result from inner ear disorders or prolonged exposure to physically demanding environments,^17^ although cognitive difficulties may stem from stress, sleep disturbances, or mental health conditions.^17–20^

Neurobehavioral symptoms are commonly categorized as vestibular, somatosensory, cognitive, and affective. Vestibular dysfunction includes dizziness, vertigo, balance problems, and spatial disorientation, which may hinder veterans’ ability to navigate dynamic environments and maintain stable employment.^11^^,^^12^ Somatosensory symptoms of numbness, tingling, chronic pain, and hypersensitivity can reduce fine motor function and coordination, limiting veterans’ ability to engage in precise activities and increasing physical discomfort, which may, in turn, cause emotional distress.^12^^,^^16^ Cognitive dysfunction, including attention, working memory, executive function, and processing speed deficits, is particularly disabling for work and school because it affects concentration, decision-making, task organization, and problem-solving skills critical to professional and academic success.^13–15^ In addition, affective dysregulation, such as mood disturbance of depression, anxiety, irritability, emotional instability, and chronic fatigue, contributes to the psychological burden of post-deployment adjustment.^17^ These emotional and cognitive challenges can result in workplace conflicts, motivational declines, social withdrawal, and diminished capacity for sustained work or structured activities.^20^^,^^21^

Given the multifactorial origins of neurobehavioral symptoms, optimizing vocational outcomes by examining their unique impact on work and other productive outcomes, independent of TBI history and in the context of other relevant mental health conditions, is crucial. Although prior research has documented differences in employment outcomes among veterans with and without neurobehavioral symptoms,^22–25^ less is known about how these symptoms relate to wider forms of productivity beyond paid work. To address this gap, this study used data from The Veteran Metrics Initiative Study^26^ to examine the association between neurobehavioral symptom groups (vestibular, somatosensory, cognitive, and affective) and veterans’ participation in both paid and unpaid productive activities. We hypothesized that post-9/11veterans experiencing neurobehavioral symptoms would have a lower likelihood of engaging in paid employment and a higher likelihood of participating in unpaid productive activities compared to veterans without symptoms.

## METHODS

### Participants

This study utilizes data from post-9/11 veterans who participated in the TVMI study, who separated within 90 days of separation from active duty or activated status in the National Guard/Reserve, and were identified in the fall of 2016.^26^ Data were collected using measures designed to assess key aspects of veterans’ post-military transition, including their paid and unpaid work involvement from 2016 to 2019.^26^ Veterans provided self-reported responses to survey items at multiple time points, but for this study, only baseline data—collected within 90 days of military separation—were analyzed.^26^ To be included in this analysis, participants were required to provide complete data for all study variables of interest.

### Measures and Variables of Interest

#### Neurobehavioral symptoms

The primary independent variable, neurobehavioral symptoms, was assessed using items from the Defense and Veterans Brain Injury Center (DVBIC)^27^ TBI Screening Tool, as implemented in the TVMI study.^26^ This screening tool is designed to identify potential deployment TBIs sustained during military service and includes neurobehavioral symptom-related questions that reflect possible outcomes of such injuries. As such, the neurobehavioral symptoms analyzed in this study—such as headaches, dizziness, memory difficulties, and irritability—are directly tied to participants’ self-reported experiences of possible military or deployment head trauma.

Veterans were first asked whether they had sustained any injuries during military service from incidents such as blasts, bullet or shrapnel wounds, vehicular accidents, or falls. Those who reported an injury were then directed to additional follow-up questions regarding symptoms they experienced. Veterans who did not report an injury were classified as having no probable deployment TBI.

All participants who reported an injury in question 1, regardless of their response to question 2, assessing injury effects, were directed to question 3, which assessed current neurobehavioral symptoms potentially associated with a possible head injury or concussion (e.g., headaches, dizziness, memory difficulties, balance problems, ringing in the ears, irritability, and sleep disturbances). Some participants endorsed neurobehavioral symptoms in Question 3 despite not meeting criteria for a probable deployment TBI (i.e., answering “yes” to Question 1 but “no” to Question 2). Consequently, not all participants reporting neurobehavioral symptoms met screening qualifications for a probable deployment TBI. Some may have experienced an undiagnosed TBI, although others may have attributed symptoms to a head or neck injury despite not meeting screening criteria.

In this study, neurobehavioral symptoms are defined as self-reported vestibular, somatosensory, cognitive, and affective symptoms among post-9/11 veterans with a history of injury during deployment during military service. This term is used instead of “post-concussive symptoms” to reflect that not all participants had a probable deployment TBI. This inclusive definition reflects the broader range of post-injury symptom experiences that may arise from diverse physical or psychological sources.

The four neurobehavioral symptom groups were categorized from the DVBIC screening tool^25^ and aligned with symptoms with established categories from the Neurobehavioral Symptom Inventory (NSI).^28^ These neurobehavioral symptom groups included vestibular (dizziness, balance issues), somatosensory (headaches, ringing in the ears), cognitive (memory problems), and affective (irritability and sleep problems). Each symptom was coded as a binary variable (0 = absent, 1 = present) based on participant responses to the DVBIC TBI Screening Tool.^26^^,^^27^ The decision to use dichotomous coding was based on the structure of the DVBIC TBI Screening Tool, which prompts participants to indicate the presence or absence of each symptom. Responses to the open-ended “Other” option were reviewed and manually coded by 2 independent coders into the appropriate symptom categories when they aligned with 1 of the 4 defined domains (vestibular, somatosensory, cognitive, and affective) within the NSI, ensuring consistency with the broader framework of neurobehavioral symptoms used in this study.

#### Productive activities

The primary dependent variable was engagement in productive activities, assessed using self-reported survey questions from the vocational domain of the Well-Being Inventory (WBI).^26^ Participants were asked about their involvement in various productive roles, including paid employment, caregiving for individuals under or over the age of 18, homemaking, volunteering, and attending school full- or part-time. Responses for each activity in the TVMI study were recorded as binary (yes/no). Because veterans could engage in multiple activities, responses were categorized into 4 mutually exclusive groups: (1) paid labor only (employed with no unpaid roles), (2) paid and unpaid labor (engaged in employment plus caregiving, volunteering, or education), (3) unpaid labor only (not employed but participating in unpaid activities), and (4) neither paid nor unpaid labor (no engagement in any productive activity).^25^

#### Covariates

*History of TBI*. The TVMI study assessed probable deployment TBI using the DVBIC screening tool.^27^ In question 1, participants reported whether they sustained injuries during their deployment because of events such as fragments, bullets, blasts, vehicular accidents (including airplane), and falls. Those who endorsed an injury in question 1 were asked whether they had experienced common TBI-related symptoms, including altered consciousness (e.g., feeling dazed, confused, or “seeing stars”), post-traumatic amnesia, loss of consciousness, or a diagnosed head injury. Respondents who answered “yes” to Questions 1 and 2 were classified as meeting criteria for a probable deployment TBI.^27^ Because the DVBIC Screening Tool explicitly asked about deployment-related injuries, we classify those who screened positive as having “probable deployment TBI.”

Additionally, pre-military TBI history was assessed using a self-reported survey question: “Did you ever experience a traumatic brain injury before military service?” (yes/no).

*Post-traumatic stress disorder* **.** Positive screen for possible PTSD was assessed using the Primary Care PTSD Screen for DSM-5 (PC-PTSD-5),^29^ a validated screening tool designed to detect probable PTSD cases. This measure consists of 5 dichotomous (yes/no) items that assess core PTSD symptoms within the past month, including re-experiencing trauma, avoidance, hypervigilance, emotional numbness, and guilt. To classify positive screen for possible PTSD, responses were dichotomized, with 4 or more affirmative responses indicating a likely PTSD diagnosis.^29^ Given the high comorbidity between TBI and PTSD and symptom overlap,^30^^,^^31^ accounting for PTSD as a covariate allowed for a clearer understanding of the independent associations between neurobehavioral symptoms and productive activity outcomes.

*Demographic covariates*. Demographic covariates included age, biological sex (Male or Female), and race and ethnicity (categorized as Hispanic or non-Hispanic; White, Black, Asian, Pacific Islander, Native U.S., and Middle Eastern). Because of small sample sizes, Pacific Islander, Native U.S., and Middle Eastern participants were combined into an “Other Race” category.

### Statistical Analysis

Descriptive statistics were used to summarize the sample characteristics. Frequencies and percentages were calculated for categorical variables, although chi-square tests were conducted to examine bivariate associations between neurobehavioral symptoms and productive activity categories.

Multinomial logistic regression models assessed the associations between neurobehavioral symptoms (vestibular, somatosensory, cognitive, and affective) and productive activity although adjusting for covariates. The assumptions of multinomial logistic regression were evaluated and met. Observations were independent, and the outcome categories were mutually exclusive. Multicollinearity was assessed using the variance inflation factor (VIF). Multicollinearity did not pose a concern in the present analyses, as the mean VIF was 1.9, and no individual predictor exhibited a VIF greater than 4. The linearity of the logit assumption did not apply, as all predictors were categorical. The sample size was sufficient for stable estimates across categories. No influential outliers were detected, and the assumption of independence of irrelevant alternatives was confirmed.

Model 1 examined the association between neurobehavioral symptoms and productive activity status, adjusting for self-reported pre-military TBI history, probable deployment TBI status, and demographic variables (age, sex, and race/ethnicity). Model 2 was further adjusted for a positive screen for possible PTSD.

Results are presented as relative risk ratios (RRs), which indicate the likelihood of being in a specific labor category relative to the reference group (i.e., individuals with neither paid nor unpaid activities). For each predictor, 95% CIs are also reported to reflect the precision of the estimates. Statistical significance was set at *P* < .05. All analyses were conducted using Stata (version 18).^32^

## RESULTS

### Descriptive Statistics

**Figure 1.**
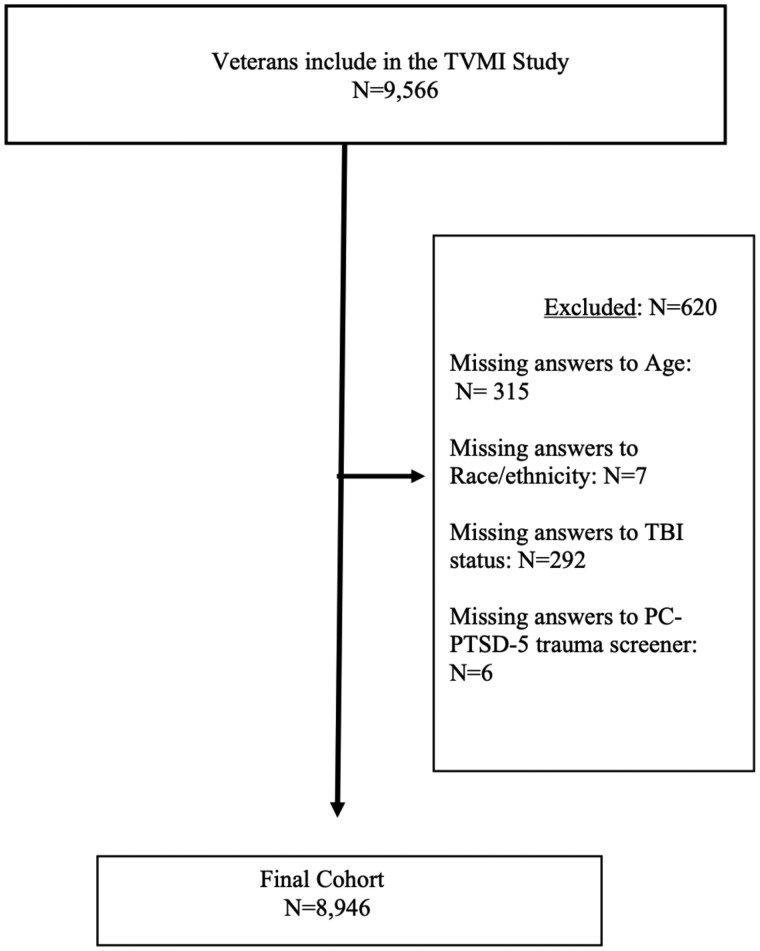
Consort diagram of inclusion and exclusion criteria.

Of the original 9,566 participants, 621 (6.5%) were excluded because of missing data. Specifically, 315 were missing age, 292 were missing TBI status, 7 were missing race/ethnicity, and 6 were missing responses on the PC-PTSD-5 trauma screener. The final analytic sample was 8,945 participants (**Figure 1**). **Table 1** shows descriptive characteristics of the sample. The majority were male and white, with a median age of 35.7 years. A minority screened positive for probable deployment TBI (14.1%) or PTSD (22.3%). **Table 1** revealed that a substantial proportion of the sample engaged in some form of productive activity, with 37.0% participating in paid labor only, 21.4% in both paid and unpaid labor, and 27.0% exclusively in unpaid labor. The remaining 14.6% of veterans reported neither paid nor unpaid labor. Symptom reporting was also notable: 18.0% experienced somatosensory symptoms, 16.9% reported affective symptoms, 11.5% reported cognitive symptoms, and 7.2% reported vestibular symptoms. The Ns for each neurobehavioral symptom represent the total number of individuals who reported that specific symptom, using a denominator of 8,945. Since participants could report multiple symptoms, these groups are not mutually exclusive.

**Table 1.** Percentage Distribution of Key Variables by Neurobehavioral Symptoms

Variable	Overall (*N* = 8,945)	Vestibular (*N* = 640)	Somatosensory (*N* = 1,605)	Cognitive (*N* = 1,032)	Affective (*N* = 1,512)	*P*-value
**Productive Activity Status**						***P* < .001**
Paid Labor Only	36.9	22.0	28.1	25.7	27.6	
Paid & Unpaid Labor	21.4	18.6	20.8	19.6	20.8	
Unpaid Labor Only	27.0	35.9	32.5	33.7	32.3	
Neither Paid nor Unpaid Labor	14.6	23.4	18.6	21.0	19.3	
**Probable Military TBI**						
Probable Military TBI (Yes)	14.1	61.4	58.7	60.3	59.2	***P* < .001**
Probable Military TBI (No)	85.9	38.6	41.3	39.7	40.8	
**Self-reported Pre-military TBI (Yes)**	8.8	20.5	18.7	19.2	17.8	***P* < .001**
**Biological Sex**						
Male	79.9	82.5	80.7	81.0	79.8	.482
Female	20.1	17.5	19.3	19.0	20.2	
**Age Group**						
18-25	8.2	7.9	8.3	8.1	7.4	.443
26-35	33.5	36.1	34.9	34.2	33.8	.312
36-45	27.3	28.3	27.8	27.1	27.2	.351
46-55	16.5	17.3	16.9	16.2	16.1	.482
56+	9.5	10.4	10.1	9.4	9.2	.561
**Race/Ethnicity**						
White	62.4	63.8	63.2	62.7	62.1	.624
Hispanic	14.5	14.1	14.9	15.1	15.0	***P* < .002**
Black	12.3	11.9	12.7	12.9	12.8	***P* < .001**
Asian	6.2	6.1	6.4	6.2	6.0	.794
Hawaiian, U.S. Indian, Other	4.6	4.1	4.3	4.0	3.7	.561
**Positive screen for possible PTSD (Yes)**	22.3	74.2	69.3	71.8	70.9	***P* < .001**

Abbreviations: PTSD, post-traumatic stress disorder; TBI, traumatic brain injury.

**Figure 2.**
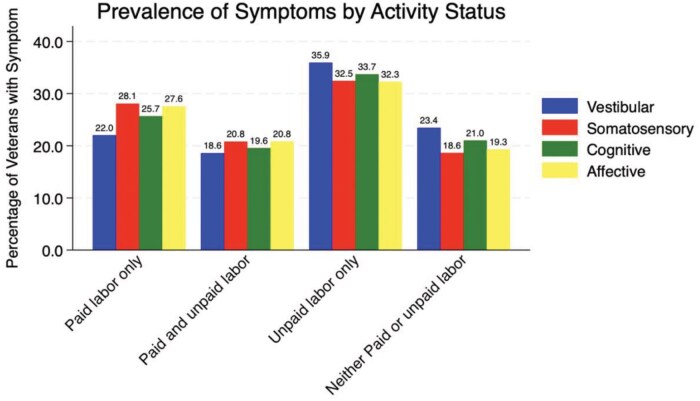
Prevalence of neurobehavior symptoms by productive activity status.

Examination of the productive activity in **Figure 2** illustrates patterns of productive activity by neurobehavioral symptom burden. Veterans in the unpaid labor only group reported the highest prevalence across all symptom categories: vestibular (35.9%), cognitive (33.7%), somatosensory (32.5%), and affective (32.3%) symptoms. In contrast, individuals in the reference group (both paid and unpaid labor) had a significantly lower prevalence of somatosensory, cognitive, and affective symptoms compared to those in the paid labor only group. Bivariate analyses using chi-square tests confirmed that all 4 symptom categories were significantly associated with productive activity status (*P* < .001).

### Multivariable Regression Analysis

#### Model 1: Neurobehavioral symptoms, Pre-Military TBI, and deployment TBI status

In Model 1, vestibular symptoms were significantly associated with a 46% lower likelihood of engaging in paid labor only compared to neither paid nor unpaid labor **(**RR = 0.54, 95% CI [0.40-0.74], *P* < .001) and a 31% lower likelihood of engaging in paid and unpaid labor (RR = 0.69, 95% CI [0.49-0.96], *P* = .027). Similarly, cognitive symptoms were associated with a 33% reduced likelihood of participating in paid labor only (RR = 0.67, 95% CI [0.49-0.91], *P* = .011). Somatosensory and affective symptoms were not significantly associated with productive activity engagement in this model.

#### Model 2: Adjusting for demographic covariates and probable PTSD

After incorporating positive screen for possible PTSD (Model 2), the risk ratios were attenuated, but the overall pattern remained. Vestibular symptoms continued to be significantly associated with a reduced likelihood of paid labor engagement (RR = 0.59, 95% CI [0.43-0.81], *P* = .001) compared to paid or unpaid labor. However, cognitive symptoms were no longer statistically significant. Instead, positive screen for possible PTSD emerged as a strong predictor of reduced paid labor engagement. Veterans screening positive for possible PTSD were 53% less likely to engage in paid labor only (RR = 0.47, 95% CI [0.39-0.56], *P* < .001) and 37% less likely to participate in paid and unpaid labor (RR = 0.63, 95% CI [0.52-0.76], *P* < .001) compared to neither paid nor unpaid labor (**Table S2**).

## DISCUSSION

This study examined the relationships between neurobehavioral symptoms and productive engagement, including paid and unpaid labor. Unexpectedly, bivariate analyses showed higher symptom prevalence among those in the unpaid labor only and neither labor groups, particularly for vestibular and cognitive symptoms. However, these associations did not hold in regression models, suggesting symptoms alone do not explain labor force disengagement (**Figure 2**).

Probable deployment TBI status remained a strong predictor of unpaid labor involvement (RR = 1.42, *P* = .009), even after adjusting for symptom burden, demographics, and other TBI history. This finding suggests who probable deployment TBI may capture factors beyond symptom burden—such as structural conditions or role expectations—that influence engagement in unpaid roles (**Table S1**).

Our findings revealed that vestibular and cognitive symptoms were associated with lower participation in paid labor only, and vestibular symptoms were associated with lower participation in paid and unpaid labor. After controlling for positive screen for possible PTSD and demographic factors, only vestibular symptoms remained a significant predictor of reduced engagement in paid labor, although cognitive symptoms were no longer statistically significant. These findings may suggest that vestibular symptoms and PTSD may have independent effects on labor force participation, whereas PTSD symptoms may account for at least part of the cognitive symptoms. The attenuation of the cognitive symptom effect after adjustment for PTSD may reflect shared variance or symptom overlap, as cognitive difficulties (e.g., impaired concentration, memory problems) are features of PTSD. It is also possible that PTSD functions as a mediating factor, contributing both to cognitive dysfunction and to broader paid labor engagement.

Our study findings emphasize the relevance of vestibular dysfunction to real-world functional limitations, which highlight its association with impaired productivity and reduced occupational functioning among affected individuals. Vestibular dysfunction—manifesting as dizziness, vertigo, and balance problems—can directly impair one’s ability to perform physical tasks safely, operate vehicles and machinery, or maintain physical orientation in office work environments.^33^^,^^34^ These challenges may be especially impactful in occupations requiring consistent physical presence, coordination, or situational awareness, creating functional barriers to productive activity engagement. For veterans, such symptoms can interfere with various productive activities, including returning to work, engaging in education or vocational training, or fulfilling caregiving and volunteer responsibilities. These findings align with prior findings from Swan et al., which demonstrated that vestibular dysfunction and dizziness are linked to broader disruptions in daily functioning among veterans with deployment-related mild or moderate/severe TBI.^35^ Wyrwa et al. also found that vestibular symptoms were associated with greater productivity restrictions, highlighting their impact on veterans’ ability to engage in employment and other productive activities.^36^ Additionally, Pogoda and colleagues used the NSI subscales and found that cognitive and affective symptoms, such as memory impairments and mood disturbances, are associated with lower employment rates among veterans.^37^

An important finding of this study is the notable prevalence of vestibular symptoms among this relatively young veteran population (median age 35.7 years). Vestibular dysfunction typically increases with age, with prevalence rates rising significantly after age 60 in civilian populations.^38^ The 7.2% prevalence of vestibular symptoms observed in our post-9/11 veteran sample suggests that military service-related factors—including blast exposure, head trauma, and other deployment-related injuries—may contribute to earlier onset of vestibular dysfunction compared to age-matched civilian populations. This elevated prevalence in a younger demographic emphasizes the unique health challenges facing younger veterans and highlights the need for early identification and intervention for vestibular symptoms during the transition period.

This study also found that screening positive for possible PTSD was associated with reduced engagement in paid labor, consistent with prior research linking PTSD symptoms to employment difficulties.^39–43^ Umucu and colleagues found that PTSD was associated with decreased social and community participation.^42^ Research by Nguyen et al. showed that veterans with PTSD are more likely to experience disruptions in social and vocational domains, which may contribute to challenges in workplace functioning.^43^ Together, these findings highlight the pervasive impact of PTSD on both employment and broader social functioning, emphasizing the need for targeted interventions to support vocational and community reintegration.

Although previous studies have primarily focused on employment as a key outcome, the current findings emphasize the significance of unpaid labor as another form of productive activity. Engaging in unpaid roles, such as caregiving or volunteering, can offer veterans meaningful opportunities for participation. Some veterans may also utilize valuable veterans’ education benefits and prioritize education after military service to advance their career goals. The association between vestibular symptoms and reduced paid labor participation may also reflect higher rates of disability compensation among veterans with more impairing symptoms. Veterans receiving disability benefits may have reduced economic pressure to engage in paid employment, allowing them to pursue education, caregiving, or other unpaid productive activities. Future research is needed to explore the factors that shape veterans’ involvement in various types of productive activities and how these activities contribute to their overall well-being.

The current findings add to this body of literature by examining multiple neurobehavioral symptoms simultaneously alongside a positive screen for possible PTSD, and by including both paid and unpaid forms of productive activity—a broader and more inclusive outcome measure than used in many previous studies. Although previous studies have emphasized cognitive and affective symptoms, our results emphasize vestibular symptoms as more strongly associated with limited paid labor participation. This contrast may reflect our broader outcome measure and simultaneous modeling of multiple symptoms, offering a more nuanced view of functional impact. This research enhances current understanding of neurobehavioral symptoms in post-9/11 veterans by highlighting the impact of vestibular symptoms and providing insight into functional outcomes through an expanded conceptualization of productivity.

Overall, these findings contribute to the growing literature on post-9/11 veterans’ reintegration experiences by demonstrating that vestibular symptoms are associated with reductions in productive activity. Future studies may explore how these relationships evolve and how treating vestibular symptoms affects productive activity. Evidence-based treatments such as Vestibular Rehabilitation Therapy (VRT)^44^ for vestibular symptoms may improve balance although helping veterans engage in paid and unpaid labor.

### Strengths and Limitations

A strength of this study is its large, diverse, and representative sample of post-9/11 veterans, enhancing generalizability. Surveying participants within 6 months of military separation also reduces recall bias in assessing productive activity during reintegration. However, several limitations should be noted.

The study relies on self-reported symptom data that were not clinically validated. Neurobehavioral symptom data were specifically drawn from self-reported responses to the DVBIC TBI screening tool, which includes items on current symptoms potentially linked to possible military injury, including probable deployment TBI. Although these items correspond to categories assessed in standardized instruments such as the NSI, the symptom measure used in this study was constructed from DVBIC screen items and does not represent a validated, stand-alone assessment tool.

Key factors influencing productive activity—such as social support, environmental context, and economic stability—were not assessed. Similarly, several potentially confounding variables were not incorporated or were not available in the dataset, including the number of lifetime TBI events, history of pre-military employment, number of deployments, whether the veteran served in a combat or non-combat role, and disability compensation. These unmeasured factors could contribute to both symptom burden and labor force participation. It is also possible that PTSD functions as a mediating factor, contributing both to the subjective experience of cognitive dysfunction and broader occupational disengagement. Future research should explore longitudinal outcomes and mechanisms linking neurobehavioral symptoms to reintegration challenges.

## CONCLUSION

This study highlights the impact of vestibular symptoms on veterans’ productive activity even after controlling for PTSD symptoms. Veterans with vestibular symptoms were less likely to engage in paid and unpaid labor. Addressing these challenges through targeted interventions may improve productive activity outcomes in veterans.

## Supplementary Material

usaf462_Supplementary_Data

## Data Availability

The data that support the findings of this study are available on request from the corresponding author, Dr Dawne Vogt.
