# Supplementary material for: Linking Neurobehavioral Symptoms to Productive Activities in Post-9/11 Veterans: A Correlational Analysis Using TVMI Data
Source: Mil Med. 2025 Oct 9;191(3-4):e737–45. doi: 10.1093/milmed/usaf462 (PMC12971021; doi:10.1093/milmed/usaf462)
Supplement: usaf462_Supplementary_Data [file usaf462_supplementary_data.zip › Supplemental Table 2.docx]

**Supplemental Table 2:** **Multivariable Models for the Outcome of Productive Activity:** Relative risk ratios from a multinomial logistic regression model predicting productive activity status as a function of probable military TBI-related neurobehavioral symptoms

| **Model** | **Paid Labor Only**  **(RR, 95% CI)** | **p-value** | **Paid and Unpaid Labor**  **(RR, 95% CI)** | **p-value** | **Unpaid Labor Only**  **(RR, 95% CI)** | **p-value** |
| --- | --- | --- | --- | --- | --- | --- |
| **Model 1** |  |  |  |  |  |  |
| **Vestibular** | 0.54 (0.40, 0.74) | <0.01 | 0.66 (0.47, 0.91) | 0.01 | 0.83 (0.63, 1.11) | 0.22 |
| **Cognitive** | 0.67 (0.49, 0.91) | 0.01 | 0.74 (0.53, 1.03) | 0.07 | 0.81 (0.58, 1.13) | 0.22 |
| **Somatosensory** | 0.96 (0.70, 1.30) | 0.81 | 1.11 (0.78, 1.57) | 0.56 | 1.09 (0.79, 1.50) | 0.61 |
| **Affective** | 0.80 (0.58, 1.10) | 0.19 | 0.96 (0.68, 1.37) | 0.83 | 0.94 (0.67, 1.33) | 0.74 |
| **Biological Sex** |  |  |  |  |  |  |
| Male | 1.71 (1.43, 2.05) | <0.001 | 1.09 (0.91, 1.31) | 0.35 | 0.63 (0.53, 0.74) | <0.001 |
| Female | 1.00 (ref) |  | 1.00 (ref) |  | 1.00 (ref) |  |
| **Age Group** |  |  |  |  |  |  |
| 18-25 | 0.72 (0.60, 0.85) | <0.001 | 0.82 (0.68, 0.99) | 0.04 | 0.94 (0.79, 1.12) | 0.49 |
| 26-35 | 1.60 (1.34, 1.90) | <0.001 | 1.20 (1.00, 1.46) | 0.05 | 0.91 (0.76, 1.09) | 0.29 |
| 36-45 | 1.17 (0.95, 1.44) | 0.14 | 0.99 (0.79, 1.24) | 0.93 | 0.56 (0.44, 0.71) | <0.001 |
| 46-55 | 0.42 (0.25, 0.68) | <0.001 | 0.45 (0.27, 0.77) | 0.003 | 0.44 (0.27, 0.72) | 0.001 |
| 56+ | 0.72 (0.60, 0.85) | <0.001 | 0.82 (0.68, 0.99) | 0.04 | 0.94 (0.79, 1.12) | 0.49 |
| **Race/Ethnicity** |  |  |  |  |  |  |
| White | 1.00 (ref) |  | 1.00 (ref) |  | 1.00 (ref) |  |
| Hispanic | 0.49 (0.41, 0.59) | <0.001 | 0.49 (0.40, 0.61) | <0.001 | 0.73 (0.61, 0.88) | 0.001 |
| Black | 0.44 (0.36, 0.53) | <0.001 | 0.46 (0.37, 0.57) | <0.001 | 0.69 (0.57, 0.84) | <0.001 |
| Asian | 0.55 (0.40, 0.75) | <0.001 | 0.58 (0.41, 0.82) | 0.002 | 1.04 (0.76, 1.42) | 0.79 |
| Hawaiian, American Indian, Other | 0.75 (0.50, 1.12) | 0.16 | 0.81 (0.52, 1.26) | 0.35 | 1.11 (0.73, 1.68) | 0.62 |
| **Model 2** |  |  |  |  |  |  |
| **Vestibular** | 0.59 (0.43, 0.81) | <0.01 | 0.69 (0.49, 0.96) | 0.03 | 0.85 (0.64, 1.14) | 0.29 |
| **Cognitive** | 0.75 (0.55, 1.03 | 0.08 | 0.70 (0.53, 1.03) | 0.07 | 0.79 (0.57, 1.09) | 0.16 |
| **Somatosensory** | 0.99 (0.72, 1.36 | 0.98 | 1.14 (0.81, 1.61) | 0.48 | 1.11 (0.81, 1.52) | 0.51 |
| **Affective** | 0.89 (0.64, 1.23) | 0.53 | 1.04 (0.74, 1.48) | 0.84 | 0.96 (0.69, 1.36) | 0.80 |
| **Biological Sex** |  |  |  |  |  |  |
| Male | 1.66 (1.39, 1.98) | <0.001 | 1.07 (0.89, 1.29) | 0.48 | 0.62 (0.53, 0.74) | <0.001 |
| Female | 1.00 (ref) |  | 1.00 (ref) |  | 1.00 (ref) |  |
| **Age Group** |  |  |  |  |  |  |
| 18-25 | 0.71 (0.59, 0.84) | <0.001 | 0.82 (0.68, 0.98) | 0.031 | 0.94 (0.79, 1.11) | 0.443 |
| 26-35 | 1.64 (1.38, 1.96) | <0.001 | 1.23 (1.02, 1.49) | 0.034 | 0.92 (0.76, 1.10) | 0.351 |
| 36-45 | 1.14 (0.93, 1.41) | 0.218 | 0.97 (0.77, 1.22) | 0.814 | 0.55 (0.44, 0.70) | <0.001 |
| 46-55 | 0.40 (0.24, 0.65) | <0.001 | 0.44 (0.26, 0.75) | 0.002 | 0.43 (0.26, 0.71) | 0.001 |
| 56+ | 0.71 (0.59, 0.84) | <0.001 | 0.82 (0.68, 0.98) | 0.031 | 0.94 (0.79, 1.11) | 0.443 |
| **Race/Ethnicity** |  |  |  |  |  |  |
| White | 1.00 (ref) |  | 1.00 (ref) |  | 1.00 (ref) |  |
| Hispanic | 0.51 (0.42, 0.61) | <0.001 | 0.50 (0.41, 0.61) | <0.001 | 0.74 (0.62, 0.89) | 0.002 |
| Black | 0.47 (0.39, 0.57) | <0.001 | 0.48 (0.39, 0.60) | <0.001 | 0.71 (0.58, 0.86) | <0.001 |
| Asian | 0.55 (0.40, 0.76) | <0.001 | 0.58 (0.41, 0.82) | 0.002 | 1.04 (0.76, 1.42) | 0.794 |
| Hawaiian, American Indian, Other | 0.79 (0.53, 1.19) | 0.258 | 0.84 (0.54, 1.31) | 0.435 | 1.13 (0.75, 1.71) | 0.561 |
| **Positive screen for possible PTSD (Yes)** | 0.47 (0.39, 0.56) | <0.001 | 0.63 (0.52, 0.76) | <0.001 | 0.79 (0.66, 0.93) | 0.006 |

**Model 1:** measures the association between activity status and neurobehavioral symptoms (vestibular, somatosensory, cognitive, and affective), adjusting for pre-military and military TBI status.

**Model 2**: measures the association between activity status and neurobehavioral symptoms (vestibular, somatosensory, cognitive, and affective), adjusting for pre-military and military TBI status and includes additional covariates: Age, gender, race/ethnicity, premilitary TBI history, probable PTSD
