# Supplementary material for: Linking Neurobehavioral Symptoms to Productive Activities in Post-9/11 Veterans: A Correlational Analysis Using TVMI Data
Source: Mil Med. 2025 Oct 9;191(3-4):e737–45. doi: 10.1093/milmed/usaf462 (PMC12971021; doi:10.1093/milmed/usaf462)
Supplement: usaf462_Supplementary_Data [file usaf462_supplementary_data.zip › Supplemental Table 1.docx]

**Supplemental Table 1**

The prevalence of symptoms associated with TBI was examined across four domains: vestibular, somatosensory, cognitive, and affective.

| **Neurobehavioral**  **Symptom Groups** | **No Military TBI**  **(n, %)** | **Military TBI  (n, %)** | **Total Prevalence  (n, %)** |
| --- | --- | --- | --- |
| **Vestibular** | 85 (1.1%) | 573 (36.2%) | 658 (7.1%) |
| **Somatosensory** | 413 (5.4%) | 1,231 (77.8%) | 1,644 (17.7%) |
| **Cognitive** | 165 (2.1%) | 896 (56.6%) | 1,061 (11.5%) |
| **Affective** | 403 (5.2%) | 1,152 (72.8%) | 1,555 (16.8%) |
